# Transcriptional complexity and roles of Fra-1/AP-1 at the uPA/Plau locus in aggressive breast cancer

**DOI:** 10.1093/nar/gku814

**Published:** 2014-09-08

**Authors:** Gabriel Moquet-Torcy, Claire Tolza, Marc Piechaczyk, Isabelle Jariel-Encontre

**Affiliations:** 1 Institut de Génétique Moléculaire de Montpellier UMR 5535, CNRS, 1919 route de Mende, 34293 Montpellier cedex 5, France; 2 Université Montpellier 2, Place Eugène Bataillon, 34095 Montpellier cedex 5, France; 3 Université Montpellier 1, 5 Bd Henry IV, 34967 Montpellier cedex 2, France

## Abstract

Plau codes for the urokinase-type plasminogen activator (uPA), critical in cancer metastasis. While the mechanisms driving its overexpression in tumorigenic processes are unknown, it is regulated by the AP-1 transcriptional complex in diverse situations. The AP-1 component Fra-1 being overexpressed in aggressive breast cancers, we have addressed its role in the overexpression of Plau in the highly metastatic breast cancer model cell line MDA-MB231 using ChIP, pharmacological and RNAi approaches. Plau transcription appears controlled by 2 AP-1 enhancers located -1.9 (ABR-1.9) and -4.1 kb (ABR-4.1) upstream of the transcription start site (TSS) of the uPA-coding mRNA, Plau-001, that bind Fra-1. Surprisingly, RNA Pol II is not recruited only at the Plau-001 TSS but also upstream in the ABR-1.9 and ABR-4.1 region. Most Pol II molecules transcribe short and unstable RNAs while tracking down toward the TSS, where there are converted into Plau-001 mRNA-productive species. Moreover, a minority of Pol II molecules transcribes a low abundance mRNA of unknown function called Plau-004 from the ABR-1.9 domain, whose expression is tempered by Fra-1. Thus, we unveil a heretofore-unsuspected transcriptional complexity at Plau in a reference metastatic breast cancer cell line with pleiotropic effects for Fra-1, providing novel information on AP-1 transcriptional action.

## INTRODUCTION

The uPA urokinase-type plasminogen activator protease is overexpressed in many cancer cells, including breast carcinomas, where it plays a crucial role in the metastatic process (1–3). Recently, guidelines from the American Society of Clinical Oncology recommended the dosage of uPA for risk assessment and treatment decision for node negative breast cancer, as high levels of uPA are associated with poor outcome (4). The transcriptional mechanisms underlying uPA gene (also known as Plau) overexpression in metastatic tumors are, however, still unknown, though a role for the AP-1 transcriptional complex in the transcriptional regulation of Plau has been proposed in diverse experimental systems (5–10).

AP-1 is an ubiquitous family of dimeric transcription factors that controls virtually all important cell decisions due to binding to so-called AP-1/TRE or CRE DNA motifs found in a wide variety of genes (11,12). AP-1 is principally made up of the members of the Fos (c-Fos, FosB, Fra-1 and Fra-2) and Jun (c-Jun, JunB and JunD) families (11,12). In contrast to Fos proteins, which must heterodimerize with transcription partners, such as the Jun proteins, the Jun proteins can also homodimerize even though their heterodimerization with Fos proteins is favored (11–16). AP-1 protein abundance and activity are controlled by intermingled transcriptional and post-transcriptional mechanisms, which are themselves regulated by intracellular signaling (17–19). Despite an abundant literature on their biological functions and the cell signaling converging onto them, the mechanisms whereby the different Fos and Jun dimers regulate transcription are surprisingly still poorly understood.

Besides its physiological roles, AP-1 is also frequently altered in cancer where it participates to tumorigenesis (14,15,20–22). Of particular interest here is the Fos family protein Fra-1, which has been shown to contribute to metastasis, invasiveness, cell motility, cell division and protection from apoptosis in diverse epithelial cancers affecting breast, ovary, thyroid, lung, bladder or colon (23–31). Its best documented pathological effects, however, concern breast carcinomas where several authors have proposed that Fra-1 might constitute a useful diagnosis marker (30) and/or a target for cancer prevention or intervention (28,31–33). More specifically, (i) Fra-1 accumulates to high levels in cell nuclei from ductal and lobular carcinomas, which contrasts with its absence in normal breast tissues and its shared cytoplasmic and nuclear distribution in a small fraction of fibroadenoma cells (30), (ii) RNAi depletion of Fra-1 in an aggressive breast cancer cell line decreases invasiveness and reverts the mesenchymal phenotype (25), (iii) Estrogen Receptor negative (ER^−^) tumors, which are more metastatic and of poorer prognosis than the Estrogen Receptor positive (ER^+^) ones, show a higher AP-1 activity essentially contributed by higher levels of hyperphosphorylated Fra-1 (25,26,32) and (iv) shRNA-mediated suppression of Fra-1 expression abrogates the metastatic potential of an aggressive human breast cancer cell line in nude mice (31).

As Fra-1 is expressed to high levels in metastatic mammary tumors where uPA (Plau) is also overexpressed and as the Plau gene was demonstrated to be an AP-1 target in other situations, we have addressed here whether Fra-1 could deregulate Plau in such cancers using RNAi-, pharmacological- and locus-wide chromatin immunoprecipitation (ChIP) analyses in a reference metastatic breast cancer cell line. Our work unveils a novel and unsuspected transcriptional complexity at the Plau locus under conditions of exacerbated and tumor aggressiveness-contributing expression.

## MATERIALS AND METHODS

### Cell culture

MDA-MB231, MCF-7, BT549, HST598, HCC38, MDA-MB157, MDA-MB436 cells were obtained from the American Type Culture Collection (Rockville, MD, USA). They were cultured in Dulbecco's modified Eagle's medium (DMEM) (MCF7, MDA-MB231, MDA-MB236, HST598) or F12/DMEM (BT549 and MDA-MB157) or RPMI (HCC38) containing 10% of fetal calf serum in a humidified 5% CO_2_ atmosphere at 37°C.

### RNAi

For RNA interference studies with siRNAs, MDA-MB231 cells were transfected with 9 nM of siRNA using the Interferin transfection system (Polyplus) according to the manufacturer's instructions. Forty-eight hours post-transfection, cells were collected and processed for RNA extraction, ChIP or immunoblotting analyses. siRNA sequences are the following: control siRNA: an equimolar mix of 5′AUGAACGUGAAUUGCUCA dTdT3′, 5′UAAGGCUAUGAAGAGAGAUACdTdT3′, 5′AUGUAUUGGCCUGUAUUAGdTdT3′ and 5′UAGCGACUAAACACAUCAAdTdT3′; Fra-1-cds siRNA: 5′CUGACUGCCACUCAUGGUGdTdT3′, siFra1-3′UTR siRNA 5′GACAGAAGGUGCCA CUUUAdTdT3′, p300 siRNA 5′UGACACAGGCAGGCUUGACUUdTdT3′, CPB siRNA 5′CGGCACAGCCUCUCAGUCAdTdT3′, PCAF siRNA 5′CGGCACAGCCUCUCAGUCAdTdT3′.

### Immunoblotting

Cells were directly lysed in 1% sodium dodecyl sulfate (SDS)-containing Laemmli sample buffer and boiled for 15 min. Proteins were fractionated by gradient (7–20%) SDS-polyacrylamide gel electrophoresis and electrotransferred onto polyvinylidene difluoride membranes (Immobilon) as previously described (34). For immunodetection, membranes were blocked overnight at 4°C in phosphate-buffered saline (PBS) containing 10% non-fat dry milk and 0.1% Tween 20, then incubated at room temperature in PBS containing 5% non-fat dry milk and 0.1% Tween 20 for 2 h with primary antibodies (described in ChIP procedures) at proper dilutions. After incubation for 2 h with horseradish peroxidase-conjugated secondary antibodies (Santa Cruz Biotechnology), proteins were detected using the Enhanced ChemoLuminescence kit from Perkin Elmer (NEL 102001EA).

### RNA extraction, polymerase chain reaction (PCR) and reverse transcription-qPCR (RT-qPCR) analyses

0.3 × 10^6^ cells were seeded in 6-well plates 48 h prior to RNA extraction, which was performed with the GenElute Mammalian Total RNA kit (Sigma - RTN70) according to the manufacturer instructions. After a 1 h treatment at 37°C with RNAse-free DNAse I (Promega), 1 μg of RNA was used for RT using 0.5 μg of either random primers (C118A), oligo-dT (C110A) from Promega or sense or antisense oligonucleotides (see Text and Supplementary Data). Reverse transcriptions were performed using the Superscript III kit (InVitroGen; 18080-044) following the manufacturer instructions. After a 10-fold dilution, 5 μl of cDNA were used for real-time PCR analysis using the Roche LightCycler 480 real-time PCR system. Data analyses were performed using the LightCycler software (Roche) and normalized with respect to invariant S26 mRNA levels. The sequences of the primers used for amplification are given in Supplementary Data S1. Semi-quantitative PCRs were performed with the Go Taq polymerase from Promega (M500B) using 30 amplification cycles and analyzed by electrophoresis through 1% agarose gels.

### ChIP and antibodies

ChIPs were performed as previously described (35). Briefly, 4 x10^6^ MDA-MB231 or 3 x 10^6^ MCF-7 cells were plated 24 h before ChIP experiments. For experiments in presence of siRNA, 2 x 10^6^ MDA-MB231 cells were transfected 48 h prior to ChIP experiments. Cell fixation with 1% of paraformaldehyde (Euromedex) for 5 min at room temperature (24°C) was stopped by addition of 125 mM glycine. Cells were then incubated for at least 2 h on ice in nuclei lysis buffer (see (35)) and then sonicated for 5 min (30 s on/off) at 4°C using the Bioruptor system from Diagenode. After sonication, absorbances at 280 nm (A_280_) of 1/100 diluted samples were measured and A_280nm_ was adjusted to 0.133 with nuclei lysis buffer. One hundred microliter were used for ChIP experiments in a final volume of 1 ml. When antibody concentrations were available from the suppliers, 3 μg of antibodies were used for ChIPs. Otherwise, 3 μl of commercial solutions were used. Antibodies against Fra-1 (sc-183X) and Cdk9 (sc-484) were from Santa-Cruz Biotechnology. Those against H3 (05–928), H3K4me_3_ (04–745), H3K9ac (07–352) and H3K36me_3_ (05–801) were from Millipore-Upstate. That against p300 (ab-14984) was from Abcam and those against Pol II (8WG16), PS5-Pol II (H14) and PS2-Pol II (H5) were from Covance. For ChIPs with H14 and H5 antibodies (which are IgMs), beads were first incubated for 20 min with 2 μg of rat anti-IgM (04-6800 InVitroGen).

### Pol II-bound RNA IP (RIP)

All buffers contained a 1X protease inhibitor cocktail (100X: 1 mg pepstatin, 1 mg leupeptin, 1 mg aprotinin, 250 mg AEBSF in 10 ml H_2_O) and 50 U/ml of RNAse•in. 4 x 10^6^ MDA-MB231 cells were plated 24 h before fixation (1% paraformaldehyde, 10 min at room temperature). After stopping fixation by addition of 125 mM glycine and incubation at room temperature for 10 min, cells were scratched and washed twice in PBS and incubated on ice in 200 μl of buffer A (5 mM PIPES pH 8.0, 85 mM KCl, 0.5% NP40) for 10 min. Nuclei were recovered by centrifugation at 2300 *g* for 5 min and washed with buffer A without NP-40. After nuclei incubation on ice in 250 μl of buffer B (1% SDS, 10 mM ethylenediaminetetraacetic acid (EDTA), 50 mM Tris-HCl pH 8.1) for 10 min, lysates were sonicated three times (Bioruptor; 30 s on/off) and centrifuged at 16,100 *g* at 4°C for 10 min. One hundred microliter of supernatant were recovered and diluted in 900 μl immunoprecipitation buffer (0.01% SDS, 1.1% Triton X-100, 1.2 mM EDTA, 167 mM NaCl, 16.7 mM Tris-HCl pH 8.1) and incubated in presence of 2 μl of anti-Pol II antibodies (8WG16, Covance) overnight at 4°C. Then, 30 μl of Dynabead protein G were added for 2 h at 4°C. After Dynabeads washing and sample elution in the same buffers as those used for ChIP, cross-links were reversed at 65°C for 2 h in 250 μl of elution buffer (1% SDS, 0.1 M NaHCO_3_, 50 U/ml RNAse•in, 200 mM NaCl). Then, 20 μl of 1 M Tris-HCl pH 6.5, 10 μl of 0.5 M EDTA and 20 μg of Proteinase K were added and samples were incubated at 42°C for 45 min. RNAs were purified using the GenElute Mammalian Total RNA kit (Sigma; RTN70) following the manufacturer instructions. Contaminating DNA was removed by incubation at 37°C with 0.05 U/μl of RNAse-free DNAse I (Promega) for 45 min. Then, purified nascent RNAs were reverse-transcribed as described above.

### Statistical analyses

Data were reported as means ± standard deviations. For calculations of means in ChIPs, the values of the signals at position −1.9 were arbitrarily referred as 1, except for H3K4me_3_ and H3K9ac, where the signal position at −1.3 was set to 1, as well as for Pol II, P-S2 and H3K36me_3_, where the signals at position 3.5 were set to 1. For calculation of means in RT-qPCR, the values of the signals for control conditions were set to 1. Significance between two conditions was assessed by the Student's paired *t*-test using the GraphPad Prism 5 software. *P-*values were considered as significant when *≤0.05 and highly significant when **≤0.01, ***≤0.0005 or ****≤0.0001.

## RESULTS

### Control of uPA gene expression by Fra-1 in MDA-MB231, BT549 and HS578T cells

**Figure 1.**
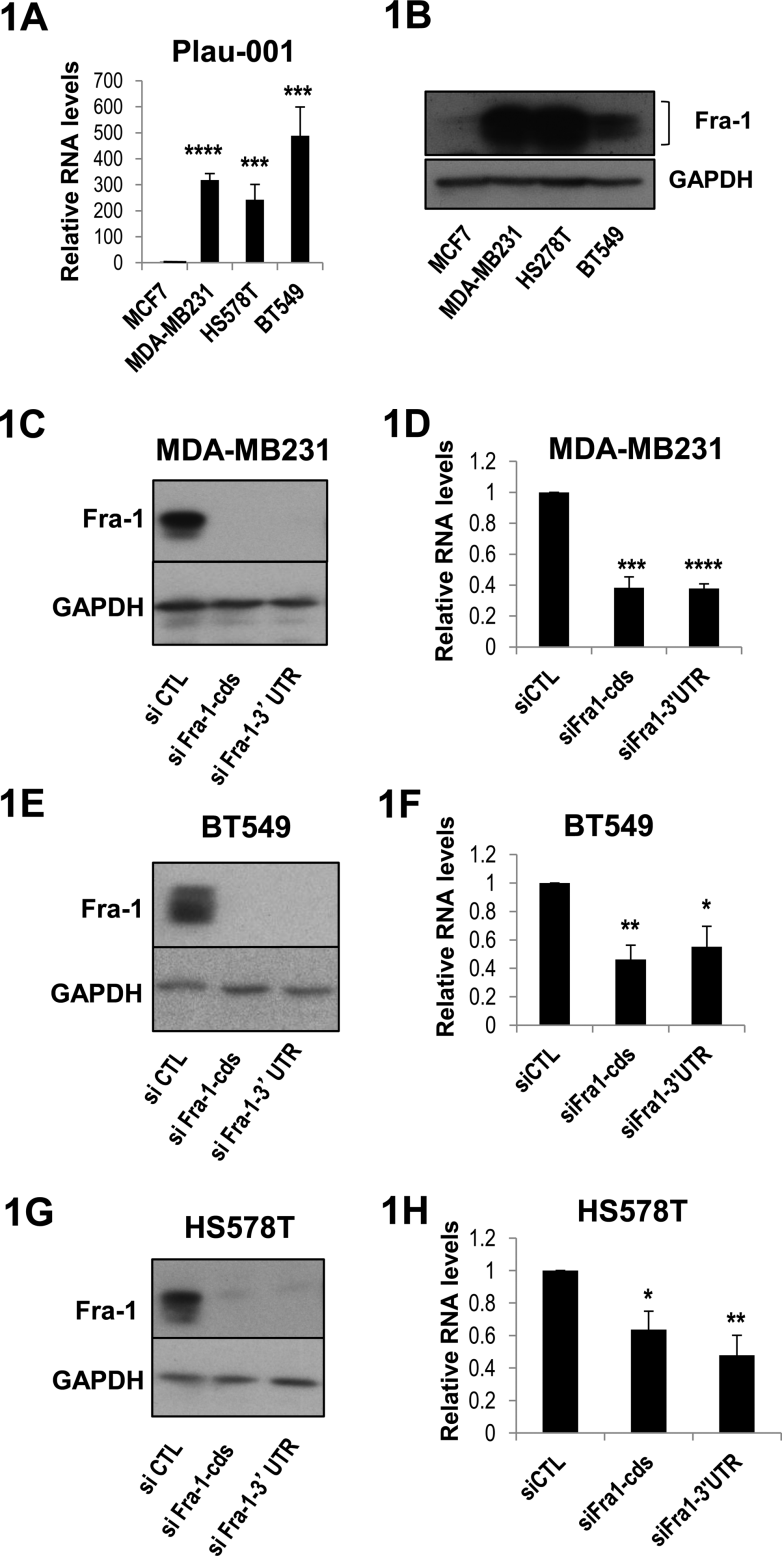
Control of uPA mRNA abundance by Fra-1 in ER^−^ breast cancer cell lines. (A) Relative abundances of Plau-001 mRNA in MCF-7, MDA-MB231, BT549, HS578T cells. mRNA abundances were assayed by RT-qPCR from total cellular RNA and normalized to S26 mRNA taken as an internal standard. The Plau-001/S26 mRNA ratio was arbitrarily set to 1 in MCF-7 cells. (B) Fra-1 protein levels in MCF-7, MDA-MB231, BT549 and HS578T cells. Fra-1 protein levels were compared by immunoblotting using a specific Fra-1 antiserum. Fra-1 is indicated by brackets. Glyceraldehyde-3-phosphate dehydrogenase (GAPDH) was taken as an electrophoresis loading control. (C, E, G) RNAi-mediated depletion of Fra-1 protein in MDA-MB231 (C), BT549 (E) and HS578T cells (G). Cells were transfected with either a control siRNA (siCTL) or siRNAs directed against the Fra-1 coding sequence (siFra-1-cds) or the 3′ unstranslated region (siFra-1–3′UTR) and protein contents were analyzed by immunoblotting with the relevant antibodies 48 h later. (D, F, H) Plau-001 mRNA reduction in siRNA-transfected MDA-MB231- (D), BT549- (F) and HS578T cells (H). Plau-001 mRNA was assayed by RT-qPCR 48 h post-siRNA transfection in the same cells as in C, E and F. Plau-001 mRNA abundance was arbitrarily set to 1 in siCTL-transfected cells for calculation of mean values. In A, D, F and H, values are the means of four independent experiments and error bars indicate standard deviations. Results of the Student's paired *t*-test are indicated on the graphs.

We first assessed whether the Plau-001 mRNA, which codes for uPA (also see below), is regulated by Fra-1. To this aim and subsequent mechanistic investigations, our study was largely based on two commonly used human breast cancer cell lines. One was the highly aggressive triple negative (ER^−^, PR^−^ and HER2^−^) MDA-MB231 cell line, which carries oncogenic mutations in KRAS and BRAF genes (36), shows enhanced PKCθ activity and accumulates high levels of Fra-1 as a result of stabilizing phosphorylations by both KRAS- and BRAF-activated Erk pathway kinases and PKCθ (26,34). The other one was the less aggressive ER^+^ MCF7 cell line, which shows no constitutive RAS/MAPK and PKCθ pathway activities and, subsequently, low Fra-1 content (25,26). In keeping with data by others (37,38), we found much higher levels (>300-fold) of Plau-001 mRNA in MDA-MB231- than in MCF-7 cells (Figure 1A) in line with run-on experiments showing much higher transcription of Plau in MDA-MB231 (39) and, consistently, with higher production of the uPA protein by MDA-MB231cells as compared to MCF-7 ones (39). Interestingly, uPA overexpression is correlated with a higher level of Fra-1 in MDA-MB231 cells (Figure 1B) as well as in two other ER negative cells lines, BT549 and HS578T (Figure 1A and B). Then, we conducted RNAi experiments in MDA-MB231 cells using two different anti-Fra-1 siRNAs to eliminate the possibility of off-target effects. One was directed to the coding region (siFra-1-cds) and the other to the 3′ UTR (siFra-1-UTR). Suppression of Fra-1 (Figure 1C) entailed a 60% reduction in Plau-001 mRNA levels, as assayed 48 h after siRNA transfection (Figure 1D). As Plau-001 mRNA is relatively stable with a half-life ≥17 h in MDA-MB231 cells (40), this values most likely largely underestimated the actual extent of uPA gene downregulation. Moreover, comparable data were obtained in the other two triple negative tumor cell lines analyzed, BT549 and HS578T (Figure 1E–H), indicating that Fra-1 is involved in Plau-001 production in the metastatic triple negative breast cancer cell lines tested.

### Binding of Fra-1 to the Plau promoter region

**Figure 2.**
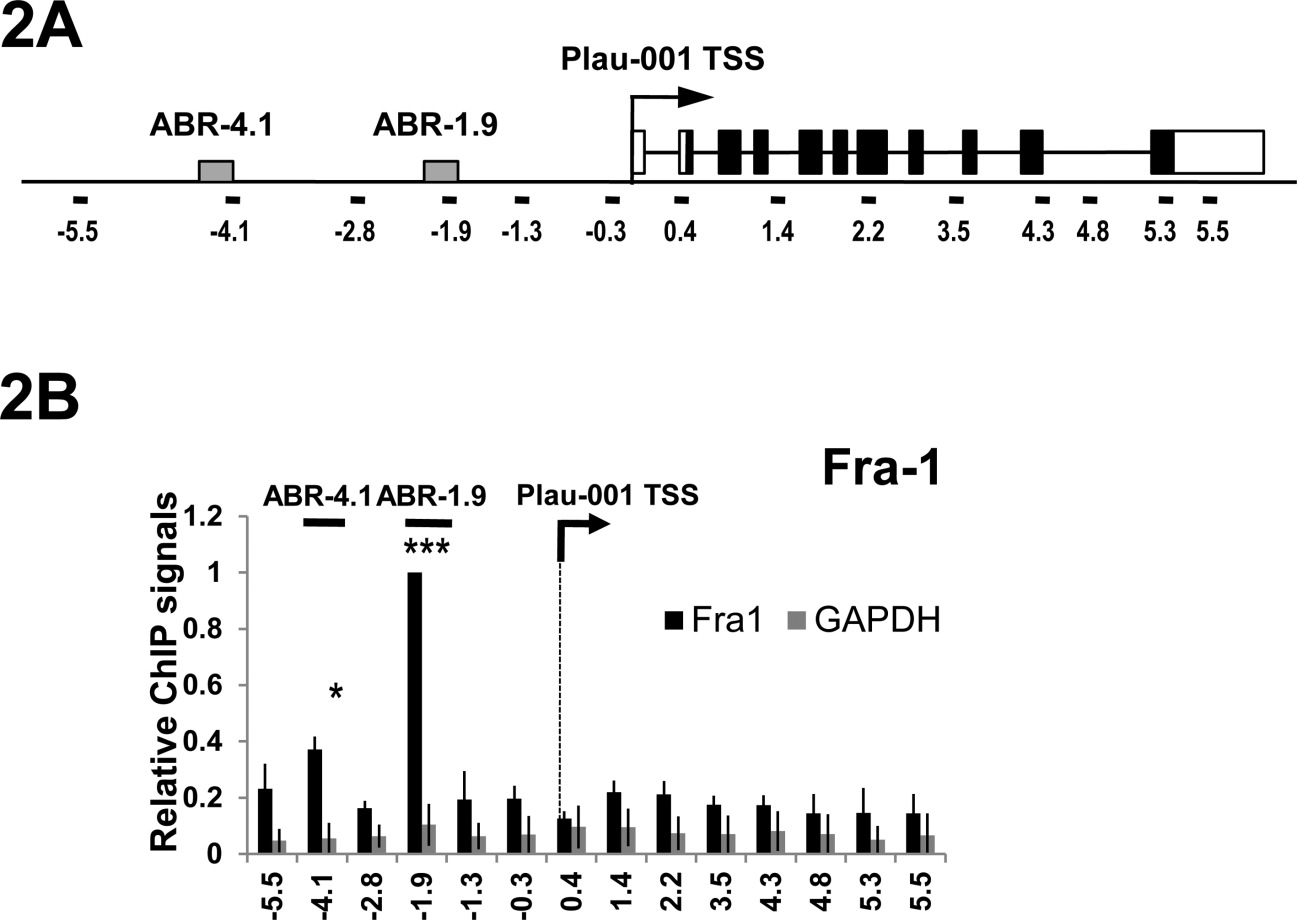
Binding of Fra-1 to the Plau locus. (A) Structure of the Plau locus. White boxes indicate Plau-001 5′UTR and 3′UTR, whereas black boxes indicate the uPA open reading frame. The arrow refers to Plau-001 TSS, as indicated in the Ensembl database. ABR-1.9 and ABR-4.1 (gray boxes) indicate Fra-1-binding regions (see text). Black lines indicate the DNA fragments (100–200 bp each) amplified by qPCR in ChIP analyses, the numbers indicating the position of their middle with respect to the TSS. (B) Fra-1 ChIP analyses. ChIP were conducted using either Fra-1-specific antibodies (black) or an anti-GAPDH antibody used as negative control (grey). Values are the means of three independent experiments and are normalized to amplicon −1.9 (containing ABR-1.9), which was arbitrarily set to 1. Primer sequences are given in Supplementary Information S1.

Bioinformatic analyses revealed the presence of at least 40 potential AP-1-binding sites (see Supplementary Data S2) at the Plau locus in the −6.5/+6.3 kb region (nucleotides are numbered herein with respect to the Plau-001 transcription start site (TSS), as positioned in the Ensembl database (http://www.ensembl.org/Homo_sapiens/Info/Index). Among them, (i) an enhancer with 2 AP-1-responsive sites has already been identified in human liver cells at −1.9 kb (8–10), (ii) an AP-1-binding site was described in mouse NIH 3T3 fibroblasts at −6.9 kb (6) in a domain homologous to the human gene −5.3 kb region and (iii) publicly available data (http://genome.ucsc.encode/) show AP-1 binding at −4.2 kb in human hepatocyte HepG2 cells. To assess which sites in the −6.5/+6.3 kb region bind Fra-1, we conducted a locus-wide ChIP analysis where 14 DNA fragments located at different positions and containing potential AP-1-binding sites were amplified (Figure 2A). These experiments were conducted in MDA-MB231 cells. They revealed a major peak of binding for Fra-1 at −1.9 kb and a minor one at −4.1 kb (Figure 2B), which were called hereafter ABR-1.9 and ABR-4.1, respectively, ABR denoting for AP-1-Binding Region.

### Chromatin signatures at the Plau locus in MDA-MB231 cells

At the level of their TSS, actively transcribed genes are generally characterized by (i) a reduced histone density (nucleosome-free regions or NFRs), (ii) histone H3 acetylated on K9 (H3K9ac) and trimethylated on K4 (H3K4me_3_) on each side of the NFR and (iii) an increased accumulation of Pol II (41,42).

**Figure 3.**
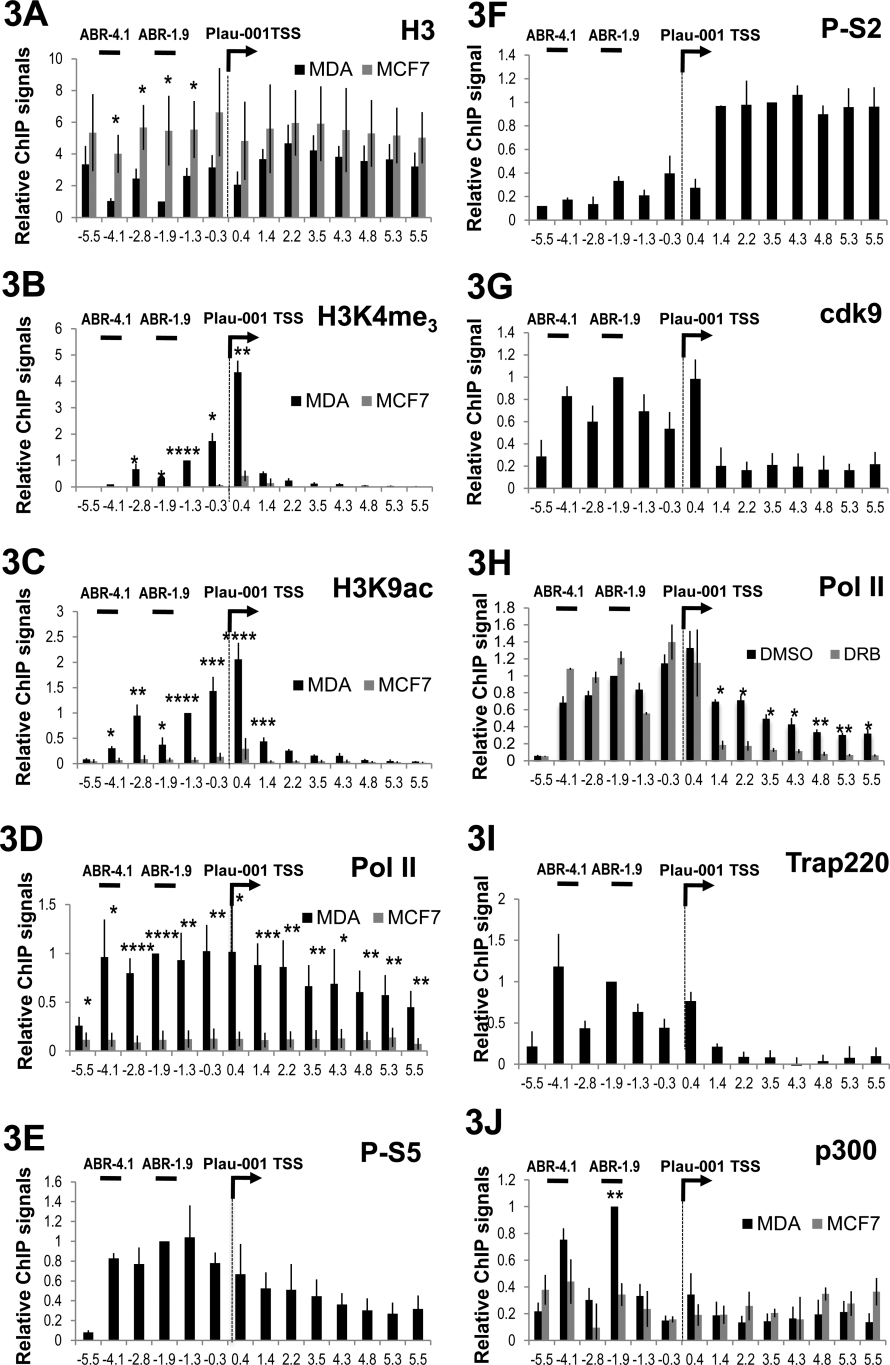
Distribution of histones, Pol II, cdk9, Trap220 and p300 on the Plau locus. ChIPs were conducted in MDA-MB231 (MDA) and MCF7 (A–D and J) cells using antibodies specific for histone H3 (A), H3K4me3 (B), H3K9ac (C), RBP1- (Pol II) (D and H), phospho-Ser5 (E), phospho-Ser2 RBP1 CTD (F), cdk9 (G), Trapp220/Med1 (I) and p300 (J). (H) ChIP conducted using an antibody against RNA Pol II in MDA-MB231 cells treated, or not, with DRB (65 μM) for 4 h. Values are the mean of at least three experiments. For calculation of means, all values were normalized to that of amplicon −1.9 in MDA-MB231 cells, which was arbitrarily set to 1, except for (i) H3K4me_3_ and H3K9ac where values were normalized to that of amplicon −1.3 set to 1 and (ii) P-S2 where values were normalized to that of amplicon 3.5 set to 1. In the case of (H), normalization was achieved with respect to control conditions. Bars indicate standard deviation. Results of the Student's paired *t*-test are indicated on the graphs.

Consistent with low transcriptional activity, ChIP analyses showed only very low levels of Pol II, H3K9ac and H3K4me_3_ in the promoter region and the absence of NFRs on the whole Plau locus in MCF-7 cells (Figure 3A–D, gray bars). In MDA-MB231 cells, we observed peaks of H3K4me_3_ and H3K9ac around the Plau-001 TSS (Figure 3B and C, black bars), in agreement with the expression of Plau-001 in these cells. Surprisingly, the most strongly reduced H3 histone density was not found at the TSS but in the domains containing ABR-1.9 and ABR-4.1 (Figure 3A, black bars). Moreover, Pol II, as assayed by ChIP targeting its RBP1 large subunit, was detected at high levels not only at the TSS (Figure 3D, black bars), as classically observed on most other genes (41,43), but also in the domain spanning ABR-4.1 to Plau-001 TSS, with a progressive decline on the body of the gene and an abrupt one upstream of ABR-4.1 (Figure 3D). Such a decline downstream of the TSS was not surprising, as it is observed on most, if not all, transcribed genes.

We also analyzed the phosphorylation of the C-terminal domain (CTD) of RPB1 in MDA-MB231 cells, as it is related to the activity and the position of Pol II on genes (44,45). The CTD consists of repeats of the YSPTSPS heptads, of which S2 and S5 are dynamically phosphorylated. S5 is predominantly phosphorylated (P-S5) by the Cdk7 subunit of the TFIIH complex. P-S5 is principally associated with the transcription-initiating form of Pol II and is typically found at TSSs and several hundred base pairs downstream (44,45). S2 is largely phosphorylated (P-S2) by the Cdk9 subunit of the P-TEFb elongation complex and is predominantly associated with the elongating form of Pol II, i.e. is usually found on the body of genes rather than in upstream promoter regions with, however, frequent overlaps with P-S5 in the 5′ parts of transcribed regions. Strikingly, the highest levels of P-S5 were not found at the Plau-001 TSS in MDA-MB231 cells (Figure 3E) but throughout the region extending from the TSS to −4.1 kb with a pattern roughly reminiscent of that of total Pol II (Figure 3D). In contrast, high levels of P-S2 were found essentially downstream of the TSS, as usually described (44–46), with a low level detected upstream of the Plau-001 start site (Figure 3F). In contrast to the distribution of P-S2, cdk9 (the P-TEFb subunit phosphorylating Pol II CTD S2) was detected on the TSS region as well as up to the −4.1 upstream region (Figure 3G). Finding little cdk9 on the gene body was consistent with the recent genome-wide analysis of Ghamari *et al.* (47) showing overlap between cdk9 and transcription-initiating Pol II (i.e. S5-P) but not with transcription-elongating Pol II (i.e. S2-P). Moreover, inhibition of Cdk9 using 5,6-dichloro-1-β-D-ribofuranosylbenzimidazole (DRB) led to a loss of Pol II signal downstream, but not upstream, of the Plau-001 TSS, consistently with the idea that S2 phosphorylation is necessary for Pol II elongation and Plau-001 mRNA transcription (Figure 3H). Indeed, DRB also led to steady-state level decrease of Plau-001 mRNA, as assayed by RT-qPCR (Supplementary Data S3).

We also assayed the distribution of the transcriptional coactivator Mediator, which usually couples Pol II to general transcription factors in transcription initiation regions (48,49) in ChIP experiments targeting its Trap220/MED1 subunit. Interestingly, Mediator covered the region spanning −4.1 kb down to the beginning of the gene body (Figure 3I) with a binding profile reminiscent to that of cdk9. This agrees with recent data showing that the MED23 Mediator subunit recruits P-TEFb via interaction with its cdk9 subunit (50). Finally, we also assessed the distribution of p300 on the Plau locus, as the association of NFRs with the presence of the histone acetyl transferase p300 constitutes a common signature of distal enhancers (41,43). While low ChIP signals were obtained in MCF-7 cells, peaks were detected at −1.9 and −4.1 kb in MDA-MB231 cells (Figure 3J).

Thus, the detection of (i) NFRs, (ii) Fra-1 and (iii) p300 by −1.9 and −4.1 kb, coupled to the previous description of an AP-1-responsive enhancer in these domains in other cell contexts (5–7,10), strongly suggested that the AP-1 enhancers for Plau-001 are ABR-1.9 and ABR-4.1. Moreover, the particular distributions of Pol II and Mediator upstream of the Plau-001 TSS raised the possibility that Pol II is mainly recruited at the ABR-1.9 and/or ABR-4.1 enhancer domains.

### Transcriptional complexity upstream of the Plau-001 TSS

An intriguing question was the role/activity of Pol II upstream of the Plau-001 TSS, which could be synthesis of sense and/or antisense RNA species and/or abortive transcription or Pol II tracking toward the TSS.

**Figure 4.**
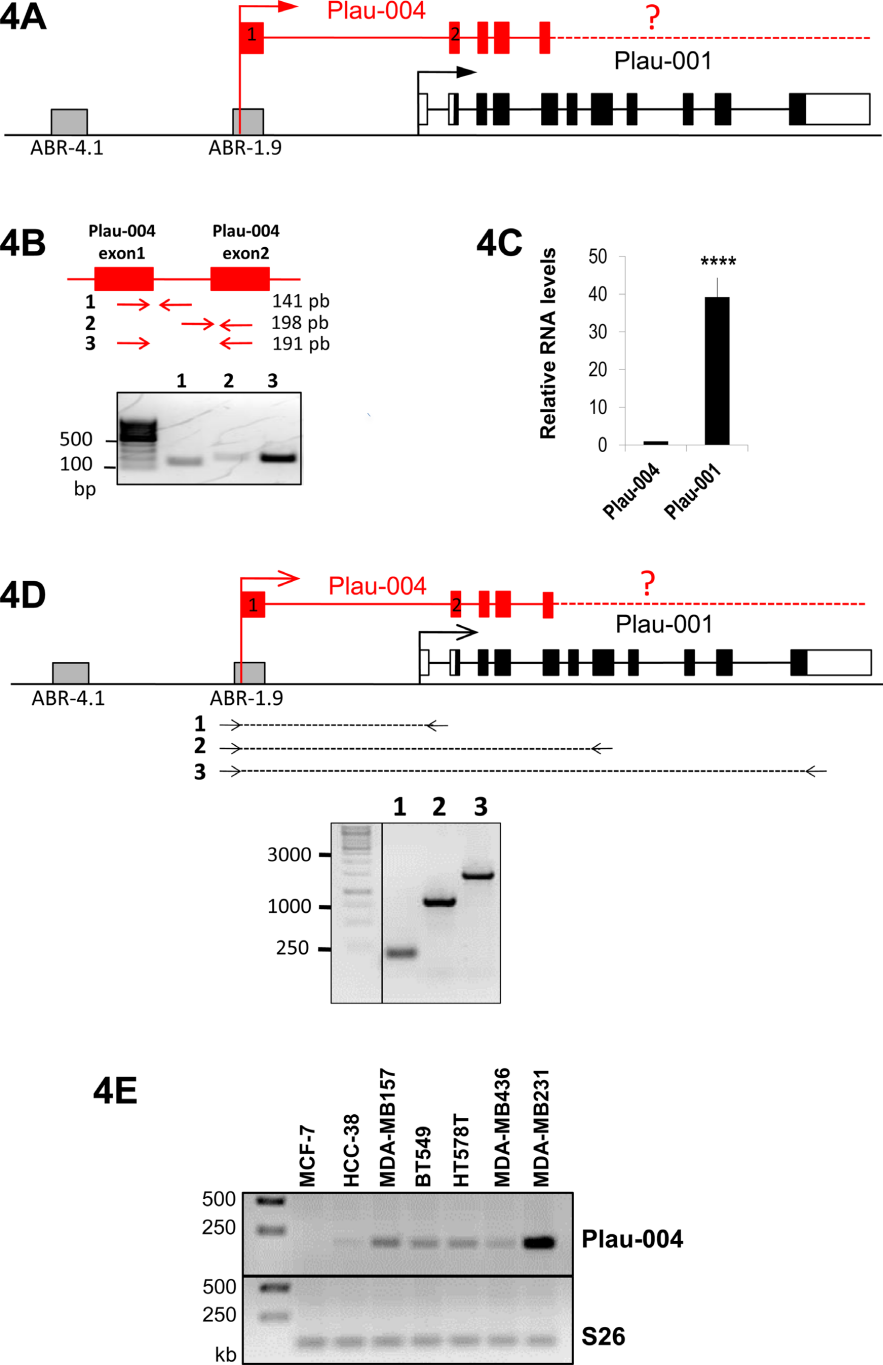
Plau-001 and Plau-004 mRNAs in MDA-MB231 cells. (A) Structure of the Plau locus: Plau-001 is depicted as in Figure 2A. Plau-004 is depicted as described in the ENSEMBL.org database. The dashed line and the question mark indicate that its 3′ end has not been characterized. (B) Plau-004 mRNA expression in MDA-MB231 cells. The sketch represents Plau-004 exon 1 and 2. Amplicon 1 and 2 correspond to non-spliced RNA, whereas amplicon 3 corresponds to spliced RNA. RT-PCR products from total MDA-MB231 cells were analyzed by agarose gel electrophoresis. (C) Relative abundances of Plau-001 and Plau-004 in MDA-MB231 cells. RNA abundances were assayed by RT-qPCR from total cellular RNA. RNA levels were normalized to that for S26 and the value for Plau-004/S26 was set to 1. (D) Plau-004 contains Plau-001 3′UTR. Reverse transcription was performed using total RNA from MDA-MB231 cells and random primers. PCRs were then carried out using a 5′ primer located in Plau-004 exon 1 and 3 downstream primers located at different positions in the uPA locus as depicted in the figure. PCR products were analyzed by agarose gel electrophoresis. (E) Expression of Plau-004 in different aggressive breast cancer cell lines. Total RNA was prepared from various breast cancer cell lines, which all express substantial amounts of Fra-1 and uPA mRNA except MCF-7. RT-PCR products corresponding to amplicon 3, as described in (B), were analyzed by agarose gel electrophoresis.

To address this, we first assessed the expression of a RNA transcript, called Plau-004, described in the Ensembl database (http://www.ensembl.org/Homo_sapiens/Gene/Summary?g=ENSG00000122861;r=10:75668935-75677255) without any indications on its function, its overall structure and/or its expression territories/conditions. The available sequence data indicate that it starts at −1.955 kb upstream of the Plau-001 TSS and that its short first exon is connected to exons 2, 3, 4 and at least part of exon 5 of Plau-001 (Figure 4A). However, there is no indication on where it terminates. RT-PCR analysis of total RNAs using amplification oligonucleotides located in Plau-004 exon 1, intron 1 and exon 2 confirmed that Plau-004 undergoes splicing (Figure 4B), whereas RT-qPCR analyses using amplification oligonucleotides located in exon 7 and 11 suggested that Plau-004 and Plau-001 share similar 3′ ends (Figure 4D), as no amplicons could be visualized when using amplification primers located downstream of the Plau-001 termination site (not shown). RTqPCR assay (Figure 4C) showed that Plau-004 is expressed in MDA-MB231 cells, where it accumulates 40-fold less than Plau-001. Finally, we could also detect Plau-004 expression in other basal-like mammary tumor cell lines but not in the less invasive MCF7 cells (Figure 4E).

**Figure 5.**
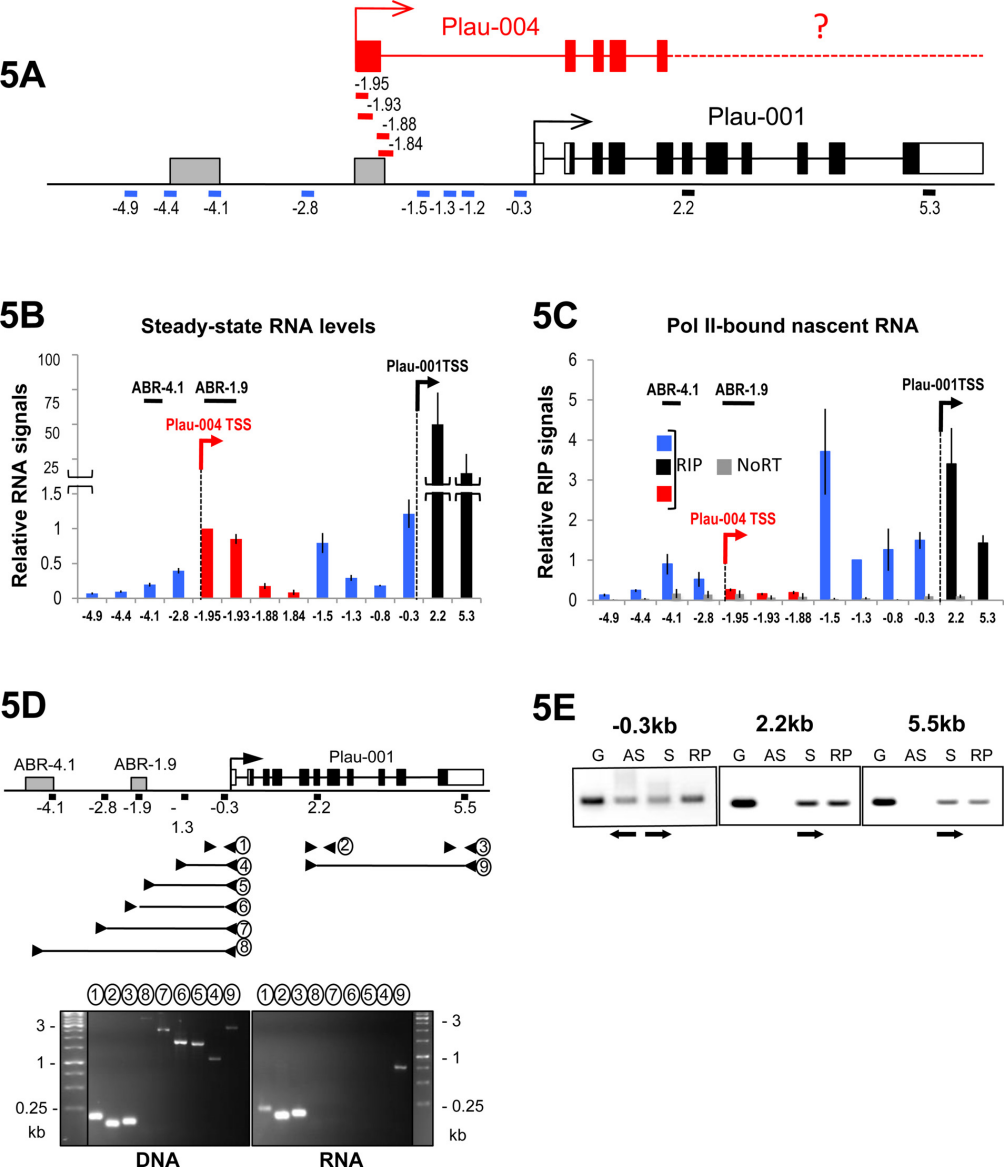
Pol II tracking at the Plau locus in MDA-MB231 cells. (A) Structure of the Plau locus*.* Black lines indicate RT-qPCR amplicons corresponding to coding sequences included in the Plau-001 mRNA. Blue lines indicate RT-qPCR amplicons corresponding to either intervening sequences not included in spliced Plau-004 mRNA or sequences lying upstream of ABR-1.9. Red lines indicate RT-qPCR amplicons whose 5′ end are located within Plau-004 exon 1 and whose 3′ ends are located within either Plau-004 exon 1 (−1.95 and −1.93 kb; i.e. amplicons included in Plau-004 mRNA) or at the beginning of Plau-004 first intervening sequence (−1.88 and −1.84 kb; i.e. amplicons not included in the spliced Plau-004 mRNA). Amplicons were all of small sizes (60–100 nt). (B) Steady-state levels of RNA transcripts upstream of Plau-001 TSS. RT-qPCRs were carried out as in Figure 4C using total RNA from MDA-MB231 cells. The color code is the same as in (A). (C) Assay of RNA Pol II-associated RNA (RIP). RNA bound to immunoprecipitated Pol II were purified as described in Materials and Methods. After reverse transcription using random primers, RT-qPCRs were conducted using the amplicons depicted in A to assess the relative abundances of nascent RNAs at different positions on the uPA locus. The color code is the same as in A and B. (D) Short RNA transcripts between ABR-1.9 and Plau-001 TSS. DNA and total RNA were prepared from MDA-MB231 cells, which was followed by reverse transcription of RNA using random primers. Amplicons of different sizes and positions were amplified by PCR as depicted in the upper panel of the figure and then analyzed by electrophoresis through agarose gels. Amplification products obtained with genomic DNA are presented in the left lower panel and permitted to validate the use of the selected amplification oligonucleotides. RT-PCR products are presented in the right lower panel. Fragments 2, 3 and 9 correspond to Plau-001 mRNA. Only fragment 1 (163 bp) could be amplified between Plau-001 and ABR-1.9. (E) Bidirectional transcription upstream of Plau-001 TSS. Total RNA from MDA-MB231 cells was prepared. Reverse transcriptions were carried out under three conditions: (i) random primers as a positive control not discriminating sense or antisense transcription, (ii) sense primers to visualize antisense transcription and (iii) antisense primers to visualize sense transcription. Oriented reverse transcriptions were initiated at positions −0.3 (upstream of Plau-001 TSS), +2.2 (within Plau-001 exon 6) or +5.5 kb (within Plau-001 3′ UTR). PCRs (30 cycles) were then performed using the same above-described sense and antisense primers plus appropriate primers allowing to generate 160, 193 and 110 bp amplicons, respectively, which were subsequently analyzed by agarose gel electrophoresis. Genomic DNA (G) was used as a positive control of amplification. S and AS refer to sense and antisense transcription, respectively, whereas RP refers to reverse transcription with random primers not discriminating sense and antisense transcription. Antisense transcription was found only upstream of Plau-001 TSS.

In a second step, we assessed the possible presence of small RNA amplicons (60–100 bp) at different places in the region upstream of the Plau-001 TSS via RT-qPCR on total MDA-MB231 cell RNA (Figure 5A). Low signals at positions −1.88 and −1.84 kb (red bars), as compared to higher signals at positions −1.95 and −1.93 kb (red bars), were consistent with splicing of the Plau-004 precursor RNA (Figures 4B and 5B). However, higher signals at position −1.5, −1.3, −0.8 and −0.3 kb (blue bars, Figure 5B) were not coherent with the idea that all Pol II molecules upstream of Plau-001 TSS were only used to generate Plau-004, since all amplicons contained within Plau-004 intron 1 were not amplified to the same level. We therefore asked whether transcription units other than that of Plau-004 may reside upstream of the Plau-001 TSS. To this aim, we analyzed transcribing-Pol II on the Plau locus by assaying nascent RNA transcripts. We used ‘RIP experiments’, consisting of Pol II ChIP experiments followed by purification and RT-qPCR assay of short (50–60 nt) Pol II-associated RNAs (Figure 5C). Strikingly, the distribution of Pol II-bound RNA was variable across the Plau locus. In particular, (i) signals were not constant in the region comprised between the Plau-004 and Plau-001 TSSs (red and blue bars), as would be expected if all RNA Pol II molecules were only transcribing Plau-004 mRNA, (ii) low levels of transcription were seen at the beginning of the Plau-004 transcription unit (first exon and beginning of intron 1; positions −1.95, −1.93 and 1.88 kb; red bars) relative to those downstream of the Plau-001 TSS (positions +2.2 and +5.3; black bars), indicating that the steady-state level difference between Plau-004 and Plau-001 RNAs (Figures 4C and 5B) is largely accounted for by differences in their transcription rates (though not excluding an additional difference in stability), (iii) higher transcriptional activity was found by ABR-4.1 and its downstream region (positions −4.1 and −2.8 kb) as compared to the beginning of the Plau-004 transcription unit (positions −1.95, −1.93 and 1.88 kb) and (iv) much higher levels of transcription were observed within Plau-004 intron 1 (positions −1.5, −1.3, −0.8 and −0.3 kb; blue bars) than by the beginning of Plau-004 transcription unit (positions −1.95, −1.93 and 1.88 kb; red bars). We note that the levels of nascent transcripts in the −1.5/−0.3 kb domain were comparable to those downstream of the Plau-001 TSS (positions +2.2 and +5.3 kb). This contrasted with the low steady-state abundance of the corresponding RNAs in total cell RNA (Figure 5B), suggesting their instability. Furthermore, we could not detect RNA longer than 250 nt in RT-PCR analysis of total RNA in the region located upstream of Plau-001 TSS (Figure 5D), suggesting essentially discontinuous transcription between ABR-1.9 and Plau-001 TSS. Moreover, oriented RT-PCR showed both sense and antisense transcription in this domain, in contrast to the exclusively sense transcription of the uPA gene body (Figure 5E).

Taken together, our data demonstrate transcriptional complexity in the Plau enhancer/promoter region. The simplest explanation is that, upstream of the Plau-001 TSS, a minority of Pol II molecules transcribe Plau-004, whereas the majority proceeds from a point located just downstream of ABR-1.9 toward the Plau-001 TSS, where they are converted into Plau-001 mRNA-productive species. Moreover, Pol II tracking is associated with production of short and unstable sense and antisense transcripts. Additionally, active transcription is observed on ABR-4.1, as has been observed on other enhancer elements.

### Effect of Fra-1 knockdown on the Plau locus

**Figure 6.**
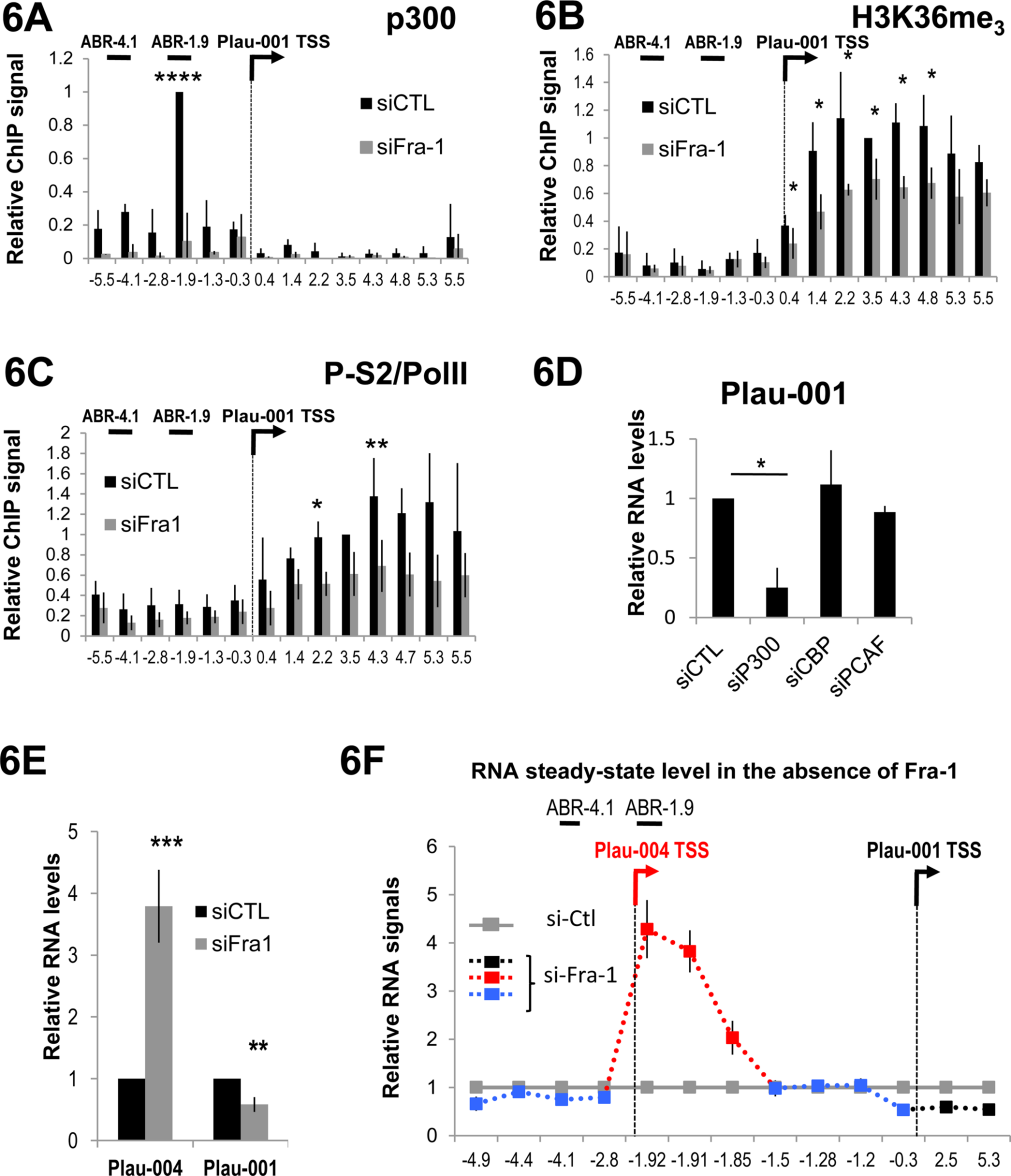
Effect of Fra-1 knockdown on the uPA locus. (A and B) Distribution of p300 and H3K36me_3_ on the Plau locus. ChIPs were conducted in MDA-MB231 cells 48 h after transfection of either a control siRNA (siCTL) or of the anti-Fra-1-cds siRNA using specific antibodies directed to p300 (A) and H3K36me_3_ (B). (C) P-S2/Pol II ratio at the Plau locus*.* ChIPs were conducted as in A and B but using antibodies directed against Pol II (RBP1 subunit) or phosphorylated Ser-2 of the RPB1 CTD. The P-S2/Pol II ratio are presented. Values are the mean of 3 (A) and 4 (B and C) experiments. For calculation of means, p300 values were normalized to that of amplicon −1.9 which was arbitrarily set to 1 in control condition. Those of H3K36me_3_ and P-S2 and Pol II were normalized to that of amplicon 3.5 set to 1 in control conditions (*P*-values are 0.052, 0.059 and 0.06 at positions +3.5, +4.7 and +5.3 kb). (D) Relative abundance of Plau-001 upon transfection of the control siRNA or of siRNA directed to p300, CBP or pCAF. Forty-eight hours after transfection, RT-qPCR were carried as in Figure 1D. Plau-001 mRNA abundance was arbitrarily set to 1 in siCTL-transfected cells for calculation of mean values. Values are the mean of three independent experiments. (E) Relative abundances of Plau-001 and Plau-004 transcripts in the absence of Fra-1*.* RT-qPCRs were carried out as in Figure 4C using total RNA from MDA-MB231 cells transfected with either a control siRNA (siCTL) or the anti-Fra-1-cds siRNA. RNA levels were normalized to that for S26 and the values for Plau-004/S26 and Plau-001/S26 in control condition were set to 1, as Plau-004 abundance is weak compared to that of Plau-001. Values are the means of three independent experiments. For (A), (B), (C), (D) and (E), bars indicate standard deviations. Results of the Student's paired *t*-test are indicated on the graphs. (F) Relative steady-state abundances of transcripts upstream and downstream of Plau-001 TSS in the absence of Fra-1*.* MDA-MB231 cells were transfected for 48 h with a control siRNA (siCTL) or with the anti-Fra-1-cds siRNA. RT-qPCR analyses were carried out as in Figure 5B. RNA levels were normalized to that of the invariant S26 mRNA. The value for each point is given relative to that of the control set to 1. The color code is the same as in Figure 5A–C. The data are the average of three independent experiments. The bars indicate standard deviations.

We then asked how Fra-1 could affect transcriptional activity at the Plau locus. In a first step, we assessed the distribution of the transcriptional machinery and histone marks in MDA-MB231 cells in which Fra-1 was depleted by RNAi (Fra-1 knockdown was verified in each experiment and was comparable to that presented in Figure 1C). No significant differences in Pol II, P-S5, Trap220, cdk9 and H3K9ac amounts and distributions were observed upon Fra-1 knockdown (not shown). In contrast, in the absence of Fra-1 using two different siRNAs, we detected a reproducible decrease in p300 recruitment upstream of the Plau-001 TSS, which was particularly pronounced on ABR-1.9 (Figure 6A and Supplementary Data S4A). This was also associated with a reduction of both H3K36me_3_ (Figure 6B) and Ser2-phosphorylation of Pol II on the gene body (Figure 6C). These last two observations were consistent with the idea that the H3K36me_3_ modification is catalyzed by a methyl transferase accompanying elongating Ser2-phosphorylated Pol II (51,52). Thus, Fra-1 does not serve to recruit Pol II, Trap or cdk9 at Plau in MDA-MB231 cells, but instead contribute to the recruitment of p300 as well as to the regulation of Pol II elongation activity. Strengthening the notion that Fra-1-dependent p300 recruitment is required for Plau-001 expression, siRNA-mediated knockdown of p300 led to a decrease in Plau-001 steady-state level (Figure 6D) associated with decreased H3K36me_3_ modification on the uPA gene body (Supplementary Data S5A). Underlining the specificity of p300 action in Plau-001 expression regulation, CBP or PCAF acetyl transferases siRNA-mediated knockdowns did not significantly alter Plau-001 expression level (Figure 6D), whereas they entailed strong decreases in MMP1 mRNA steady-state levels (Supplementary Data S5B).

Lastly, we asked whether Fra-1 knockdown by RNAi would alter Plau-004 expression and Pol II tracking upstream of the Plau-001 TSS. An RTqPCR assay similar to that used in Figure 4C showed that, upon Fra-1 depletion, Plau-004 abundance increased 4-fold (Figure 6E), whereas that of Plau-001 was reduced by 2-fold (Figures 6E and 1D). This indicated an additional role for Fra-1 at the Plau locus, i.e. repression of Plau-004 transcription. Next, we measured whether Pol II tracking-generated RNAs vary like Plau-001 and Plau-004 upon Fra-1 depletion, using the same RTqPCR assay as that in Figure 5B. Consistent with the data of Figures 6E and 1D, the abundance of small amplicons derived from Plau-004 increased 4-fold (amplicons −1.92, −1.91 and −1.85 kb; in red) while that of those derived from Plau-001 (amplicons +2.2 and +5.3; in black) decreased by 2-fold (Figure 6F). Little variation was observed for amplicons derived from tracking RNAs (amplicons −1.5, −1.3 and −0.3). The fact that Plau-004 transcript levels, but not those of Pol II tracking-generated ones, vary upon Fra-1 depletion RNAs further stressed the different nature of these RNA species.

In summary, these data show antagonistic effects of Fra-1 on Plau-001 and Plau-004 transcription. They also confirm the notion that Pol II molecules responsible for Plau-001 synthesis first track over the ABR-1.9/Plau-001 TSS domain before being converted in Plau-001 mRNA-productive forms.

## DISCUSSION

Highly aggressive triple negative breast cancers are a leading cause of death by cancer among women and invasiveness of such cancers is linked to overexpression of metastasis-promoting genes, such as Plau (1–3). Deciphering the molecular mechanisms underlying their upregulation is, therefore, of utmost importance to understand the biology of these tumors and to identify pathways that might be targeted by future therapies. Plau being responsive to AP-1 in diverse situations (5–10), we have asked here whether it could be a target of Fra-1, as Fra-1, a Fos family member, is recurrently overexpressed in aggressive mammary tumors where it contributes to the tumor phenotype (14,20,31,32). Our herein data reveal unsuspected transcriptional complexity at the Plau locus in MDA-MB231 cells with two overlapping transcription units generating two different RNAs (Plau-001 and Plau-004) coupled to an unusual Pol II tracking mechanisms from an upstream enhancer to the Plau-001 TSS. Moreover, they reveal antagonistic functions of Fra-1 on the transcription of Plau-001 and Plau-004.

### Transcription of Plau-001

Our data indicate that Fra-1 is recruited at two AP-1 binding sites showing typical additional enhancer signatures (41), i.e. low nucleosome density and binding of p300. One enhancer (ABR-1.9) is located 1.9 kb upstream of Plau-001 TSS, as already described in other cell contexts (8–10). The other one (ABR-4.1) is located 4.1 kb upstream of Plau-001 TSS and has been reported in a publicly available database (http://genome.ucsc.encode/) to be an AP-1-binding site in human hepatocytic HepG2 cells. However, in its present state, our study does not exclude that other AP-1-binding sites located further upstream may also participate to uPA (de)regulation. Strikingly, the distribution of Pol II (as well as that of Mediator) on the Plau locus strongly suggests that Pol II is not recruited at the level of Plau-001 TSS in MDA-MB231 cells but rather in the TSS upstream region up to ABR-4.1. Moreover, RIP experiments and RTqPCR assays on total cell RNA indicate transcription in the −4.1/TSS domain with stronger transcription downstream of ABR-1.9 in the -1.5/TSS region. Our data also support a model whereby Pol II tracks down a site located downstream of ABR-1.9 toward the Plau-001 TSS while transcribing short and unstable RNA from both DNA strands.

Transcription in the Plau enhancer region and upstream of the Plau-001 TSS is to be considered with two recent sets of work. First, large-scale ChIP analyses have shown co-association of Pol II and p300/CBP at numerous enhancers (41,43,53,54). Such an association of Pol II with enhancers is not a general indirect consequence of enhancer/TSS interaction revealed by chromatin cross-linking in ChIP assays. Rather, high-throughput RNA-seq analyses have demonstrated production of so-called eRNAs at Pol II-associated enhancers that can reside very far away from their cognate genes (43). These eRNAs, the role of which is still unknown, are <2000 nucleotide long and are sense and antisense polyA^−^ species (43). Second, Pol II tracking from enhancers to TSSs has been described for a few genes that include those for prostate-specific antigen (PSA) (55), β-globin (56), ϵ-globin (57) and 11β-hydroxysteroid dehydrogenase type 2 (58). In these cases, both short sense and antisense RNA species were detected. However, the function(s) of these RNAs remain(s) to be elucidated, although the possibility that some of them are produced erroneously as a natural function of Pol II cannot be excluded. Thus, our study of Plau in MDA-MB231 cells extends the notion that certain enhancers can be, not only sites of Pol II recruitment, but also sites of active transcription.

The fact that we detect short unstable RNAs between ABR-1.9 and Plau-001 TSS is reminiscent of the above-mentioned cases of Pol II tracking (55–58), although the fraction of Pol II actively transcribing during tracking has not been addressed in the latter situations. It is therefore of note that nascent RNAs are of rather equivalent abundance upstream and downstream of Plau-001 TSS. This implies that most, if not all, Pol II molecules that transcribe Plau-001 have most likely previously tracked between the enhancer and the TSS. Interestingly, data obtained in the K562 cell line and available in the UCSC databank reveal low amounts of PolyA^+^ nuclear RNA (>200 bp) transcribed in the −1.5 kb/TSS region, suggesting that tracking during uPA gene expression may not be restricted to MDA-MB231 cells. It is also worth considering that tracking Pol II molecules are essentially phosphorylated on CTD Ser5, whereas Plau-001-generating ones are phosphorylated on Ser2. A limiting step for converting tracking Pol II into a Plau-001 productive form may therefore reside in a change in the P-S5 versus P-S2 ratio in the TSS region.

We show here that Fra-1 binding is required for p300 recruitment at the Plau locus and exclude roles for the other histone acetyl transferases PCAF and CBP in the regulation of Plau-001 mRNA in MDA-MB231 cells. In agreement with a direct recruitment of p300 by Fra-1, *in vivo* interaction between Fra-1 and p300 proteins has already been reported (59) and Fos/Jun dimers can directly recruit p300 at target AP-1 sites in *in vitro* reconstituted chromatinized environments, which constitutes a prerequisite for AP-1-dependent transcription of human papilloma virus genes (60). Moreover, preliminary data indicate that it may concern Fra-1 targets other than Plau, as p300 recruitment at an intronic AP-1 enhancer of the AXL gene (29) depends on the presence of Fra-1 in MDA-MB231 cells (see Supplementary Data S6).

An intringuing point is that our ChIP experiments show that cdk9 distribution on the Plau locus overlaps with transcription-initiating Pol II (P-S5 Pol II) rather than with elongating Pol II (P-S2 Pol II), despite the fact that cdk9 has been described for phosphorylating Pol II on Ser 2 of the CTD of its RPB1 subunit. Such an apparent discrepancy has already been reported by Ghamari *et al.* (47). Using a combination of ChIP-Seq, immunostaining and live cell imaging experiments, these authors showed, on one hand, colocalization of cdk9 and P-S5 Pol II at the 5′ end of genes engaged in transcription factories and, on the other hand, absence of colocalization of P-S2 Pol II with both P-S5 Pol II and cdk9, suggesting that transcription initiation and elongation occur in different compartments of the nucleus. As they also observed that transcription factories are highly dynamic in exchanging cdk9, they also proposed that P-S2 Pol II moves out of transcription factories to achieve its elongation function after having been phosphorylated by cdk9. Although these conclusions were drawn from experiments involving large-scale studies, further work is still necessary to demonstrate whether this scenario applies to Plau.

As the deletion of Fra-1 by RNAi results also in a decrease of both P-S2 Pol II and H3K36me_3_ on the Plau gene body, it is plausible that Fra-1-recruited p300 contributes to P-TEFb activation and subsequent formation of the P-S2 elongating form of Pol II at the Plau locus. Indeed, it has been described that a large fraction of P-TEFb (cdk9/cycT1) is inactivated in cells by an inhibitory ribonucleoprotein complex and acetylation of cycT1 by p300 is required for inhibitor release and subsequent activation of Cdk9 (61). Current work is aiming at testing this hypothesis at the level of Plau.

Plau is an inducible gene in various instances (stimulation of cells with TPA, FGF-2 or IL-1 or through induction of cytoskeleton reorganization) where induction is dependent on AP-1 and, most often, its c-Jun component (5,6,62). In PC3 prostate cancer cells, where it is expressed constitutively in a c-Jun-dependent manner, the Plau locus has been reported to form a chromatin loop juxtaposing the AP-1 site-containing enhancer- and the Plau-001 TSS domain (7). This led the authors to hypothesize that looping might be essential for activation of Pol II recruited at the TSS by c-Jun and p300 recruited at the AP-1 enhancer. This conclusion was largely based on the facts that, (i) ChIP assays using cross-linked and fragmented chromatin revealed the association of the Sp1 and c-Jun transcription factors, as well as of p300 and the elongating form (but not the initiating one) of Pol II not only with the minimal promoter region of the uPA gene but also with the enhancer and (ii) RT-PCR assays showed RNA species at the level of the promoter but not at that of the enhancer. However, arguing that looping may not be critical (and, possibly hardly occurs) in MDA-MB231 cells, the distribution of Fra-1, p300, H3K9Ac and H3K4me_3_ was particularly unbalanced at the Plau locus with much higher signals of the former two by ABR-1.9 and much higher signals of the latter two by Plau-001 TSS in our ChIP experiments. Additionally, Ser5-phosphorylated Pol II was more abundant upstream of Plau-001 TSS, whereas Ser2-phosphorylated Pol II was more abundant downstream of it. It ensues from these observations that the mechanisms governing the 3D organization of the gene, and therefore of its transcription modalities, may differ depending on the situation. Whether this relies on the different AP-1 dimers requires further investigation.

Fra-1 does not act on its own but must heterodimerize with other AP-1-constituting proteins to exert its transcription factor activity. Preliminary ChIP analyses revealed the presence of c-Jun, JunB and JunD at ABR-1.9 and ABR-4.1 (Supplementary Data S7). They also showed the presence of Fra-2, which is also overexpressed in MDA-MB231 cells (whereas c-Fos and FosB are not expressed to detectable levels), at the same sites (Supplementary Data S7). This raises the interesting issues as to whether Fra-1 and Fra-2 exert complementary and/or redundant functions on exacerbated uPA gene expression and are present at the same time on Plau or undergo binding cycles one after the other. Another issue is whether they preferentially heterodimerize with specific member(s) of the Jun family members or indifferently dimerize with all three Jun proteins to stimulate uPA transcription. It must also be considered that, even though heterodimerization is favored, the Jun proteins can also homodimerize. Binding cycles at ABR-1.9 and ABR-4.1 might therefore not just concern Fra-1/2:Jun but also Jun:Jun dimers. Further work is necessary to elucidate these points.

### Transcription of Plau-004

Plau-004 RNA is detectable in MDA-MB231 cells, as well as in other aggressive breast cancer cell lines. It is, however, expressed at very low levels, not only in comparison to Plau-001 in total, but also in comparison to nascent RNAs in the region where Pol II tracks before reaching Plau-001 TSS. Its initiation site is located in the ABR-1.9 region with a first exon spanning nucleotides −1980 to −1862. Moreover, RT-PCR analyses indicate that its description found in the Ensembl database is incomplete (reported end in exon 5 of Plau-001) and that, most probably, Plau-004 ends like Plau-001. An important question will now be to identify its biological function(s).

Interestingly, our data indicate that Fra-1 operates as a transcriptional activator for Plau-001 but has a negative effect on Plau-004 synthesis in MDA-MB231 cells. As we observed active transcription between ABR-4.1 and ABR-1.9 in our RIP experiments, an interesting possibility would be that the Pol II molecules recruited by ABR-4.1 would track down to the Plau-004 TSS where they could be partially halted by ABR-1.9-bound Fra-1. Another possibility might be a competition between Plau-001 and Plau-004 TSSs for transcription initiating factors, which would be tipped toward Plau-001 when Fra-1 is present. Further work is, however, still required to discriminate between these hypotheses, as well as to identify the mechanisms whereby Pol II molecules are selected in the ABR-1.9 region to give rise to Plau-004 or for tracking toward Plau-001 TSS to eventually synthesize Plau-001.

Thus, our work reveals a heretofore-unsuspected transcriptional complexity in the enhancer/promoter region of Plau in breast cancer cells where Fra-1 is overexpressed. In particular, we unveiled a Pol II tracking mechanism upstream of Plau-001 TSS. Important questions to solve will now be to establish whether the same occurs in other cell context and/or depend on the nature of the AP-1 dimers recruited at the Plau enhancers and to identify the mechanisms that are responsible for conversion of tracking Pol II in an mRNA productive form at the Plau-001 TSS.

## SUPPLEMENTARY DATA

Supplementary Data are available at NAR Online.

SUPPLEMENTARY DATA
