# Supplementary material for: Transcriptional complexity and roles of Fra-1/AP-1 at the uPA/Plau locus in aggressive breast cancer
Source: Nucleic Acids Res. 2014 Sep 8;42(17):11011–24. doi: 10.1093/nar/gku814 (PMC4176185; doi:10.1093/nar/gku814)
Supplement: SUPPLEMENTARY DATA [file supp_42_17_11011__index.html]

Transcriptional complexity and roles of Fra-1/AP-1 at the uPA/Plau locus in aggressive breast cancer — Transcriptional complexity and roles of Fra-1/AP-1 at the uPA/Plau locus in aggressive breast cancer — SUPPLEMENTARY DATA 

# Transcriptional complexity and roles of Fra-1/AP-1 at the uPA/Plau locus in aggressive breast cancer

## SUPPLEMENTARY DATA

**Files in this Data Supplement:**

- SUPPLEMENTARY DATA
