# Supplementary material for: Transcriptional complexity and roles of Fra-1/AP-1 at the uPA/Plau locus in aggressive breast cancer
Source: Nucleic Acids Res. 2014 Sep 8;42(17):11011–24. doi: 10.1093/nar/gku814 (PMC4176185; doi:10.1093/nar/gku814)
Supplement: SUPPLEMENTARY DATA [file supp_gku814_nar-03124-v-2013-File008.pdf]

Supplementary Data S1
primer sequences used for PCR and qPCR

position	name	orientation	sequence 5'-3'
-5.5	MP4034	sense	TCCAAACCCTCCTCAGAGGCTCAGC
	MP4035	antisense	TCCCTTTTCCCGATCACCATCCATC
-4.1	MP4029	sense	TCAGTGCCTGCCGTTGCCAGC
	MP4031	antisense	TGCCGTTGCCAGCTGGCTTCA
-2.8	MP3390	sense	CCCTCCCTTCACAGCGTTCTG
	MP3391	antisense	GATGAGCAGCAAAGCCAGCAC
-1.9	MP3169	sense	AAGGGGAGAAAGGGTGTCACGCTT
	MP3170	antisense	CAGCCTGAGGACTTACCCCGAAAC
-1.3	MP4132	sense	CGAGTCCTACTGGGTTCAAAATGAC
	MP4133	antisense	ACACACACACACACACACGCATCTC
-0.3	MP3394	sense	CCAGCGGGGAGAAGTTGCAGA
	MP3395	antisense	TCTCCGTGCTTCCTCCCATGC
0.4	MP3396	sense	CCCAGAGAGGAGAGAGACAGC
	MP3397	antisense	GACCAGGACGCAGAGAAGCAG
1.4	MP3452	sense	GTGAGGTGGGGTTGGAAGGAG
	MP3453	antisense	AGGGTTACACCATGTTGTCCAGG
2.2	MP3400	sense	TGAGGGGAGGAGGCAGGGAAG
	MP3401	antisense	CACCCCACTGTCATCAGCAGG
3.5	MP3402	sense	GCCTAACTACCTCAGCTGGGA
	MP3403	antisense	TAGGTCAGTCATCCAGGGAGG
4.3	MP3404	sense	CCCCACTACTACGGCTCTGAA
	MP3405	antisense	CACCCTTAAGCTGGCTGGAAC
4.8	MP3406	sense	GGTGGGGAGGAGAACTAGGGT
	MP3407	antisense	CATGAGCCACTGTGCCTGACG
5.3	MP3163	sense	TGTTCCCTCCAAGGCCGCATGA
	MP3164	antisense	TGGTGTGACTGCGGATCCAGGGTAA
5.5	MP3408	sense	CCCGCTTTCTTGCTGGTTGTC
	MP3409	antisense	CTGGTGGGTGGCACAGGCAAA

Primers used in Figure 2B, Figure 3 all panels, Figure 5E and Figures 6A, 6B, 6C

amplicon	name	orientation	sequence 5'-3'
1	MP3169	sense	AAGGGGAGAAAGGGTGTCACGCTT
	MP3170	antisense	GCAGCCTGAGGACTTACCCCGAAA
2	MP3396	sense	CCCAGAGAGGAGAGAGACAGC
	MP3397	antisense	GACCAGGACGCAGAGAAGCAG
3	MP3169	sense	AAGGGGAGAAAGGGTGTCACGCTT
	MP3397	antisense	GACCAGGACGCAGAGAAGCAG

Primers used in Figure 4B

amplicon	name	orientation	sequence 5'-3'
Plau-004	MP3169	sense	AAGGGGAGAAAGGGTGTCACGCTT
	MP3170	antisense	CAGCCTGAGGACTTACCCCGAAAC
Plau-001	MP3292	sense	ATTGGGGGAGAATTCACCACCATC
	MP3293	antisense	CTGATGAGGCTGCCTCCACACACGT

Primers used in Figures 1A, 1D, 1F, 1H, in Figure 2G, in Figures 4C, 4E and in Figures 6D, 6E

position	name	orientation	sequence 5'-3'
-4.9	MP4249	sense	CACCCAACATGGTAACCAGAACTCT
	MP4250	antisense	CTCACCAGGAACCAAAGTTAGGCCC
-4.4	MP4251	sense	AGGCAGAAAAAGAGTGAGGAAGGTT
	MP4252	antisense	CATGAAAGGGGAACTAAAGCTGGTC
-4.1	MP4253	sense	CCGTACCTCCTTCCCAGAACTGTTG
	MP4254	antisense	AAGCGCCTCTTTCCCTCCTACACAT
-2.8	MP4257	sense	CCCTCCCTTCACAGCGTTCTGTCTT
	MP4258	antisense	CCCAGCGTCTTCTGATCCTTGTTCC
-1.95	MP3169	sense	AAGGGGAGAAAGGGTGTCACGCTT
	MP4238	antisense	GCTCCCCTAGCAGCTCTCATGACTC
-1.93	MP3169	sense	AAGGGGAGAAAGGGTGTCACGCTT
	MP4239	antisense	CAGGAAATTCCCAGGGACCGTCATG
-1.88	MP3998	sense	TGAATCATGACGGTCCCTGGG
	MP4240	antisense	CCTGAGGACTTACCCCGAAACTCCC
-1.84	MP3998	sense	TGAATCATGACGGTCCCTGGG
	MP4241	antisense	CAGTTTTGTTTGGATTTGAGAACCC
-1.5	MP4290	sense	GAGCCTTCCTCCTTCCTACCTTCCT
	MP4291	antisense	TGGGTCCACCTGAGAGTGACAGAAG
-1.3	MP4263	sense	CCTGACACTAGGGGAAAGAGATGGG
	MP4264	antisense	GCACACTTGGAGGAACATAGCAGGG
-0.8	MP4265	sense	TCCCAAAAGACCCGTTAACACTTCA
	MP4266	antisense	TACAGGATTGTGGGAACAAAGTCCT
-0.3	MP4267	sense	ACTGATTAGAGGACCCCAGGAGGCT
	MP4268	antisense	AGTCCCCGAATTGCAGAGAGGAGAG
2.2	MP3400	sense	TGAGGGGAGGAGGCAGGGAAG
	MP3401	antisense	CACCCCACTGTCATCAGCAGG
5.3	MP3163	sense	TGTTCCCTCCAAGGCCGCATGA
	MP3164	antisense	TGGTGTGACTGCGGATCCAGGGTAA

Primers used in Figures 5B, 5C and in Figure 6F

amplicon	name	orientation	sequence 5'-3'
1	MP3394	sense	CCAGCGGGGAGAAGTTGCAGA
	MP3395	antisense	TCTCCGTGCTTCCTCCCATGC
2	MP3400	sense	TGAGGGGAGGAGGCAGGGAAG
	MP3401	antisense	CACCCCACTGTCATCAGCAGG
3	MP3408	sense	CCCGCTTTCTTGCTGGTTGTC
	MP3409	antisense	CTGGTGGGTGGCACAGGCAAA
4	MP4132	sense	CGAGTCCTACTGGGTTCAAAATGAC
	MP3395	antisense	TCTCCGTGCTTCCTCCCATGC
5	MP3169	sense	GTCCTCAGCAATCAGCATGACAGCC
	MP3395	antisense	TCTCCGTGCTTCCTCCCATGC
6	MP3167	sense	AAGGGGAGAAAGGGTGTCACGCTTC
	MP3395	antisense	TCTCCGTGCTTCCTCCCATGC
7	MP3390	sense	CCCTCCCTTCACAGCGTTCTG
	MP3395	antisense	TCTCCGTGCTTCCTCCCATGC
8	MP4029	sense	TCAGTGCCTGCCGTTGCCAGC
	MP3395	antisense	TCTCCGTGCTTCCTCCCATGC
9	MP3400	sense	TGAGGGGAGGAGGCAGGGAAG
	MP3409	antisense	CTGGTGGGTGGCACAGGCAAA

Primers used in Figure 5D

amplicon	name	orientation	sequence 5'-3'
1	MP3169	sense	AAGGGGAGAAAGGGTGTCACGCTT
	MP3397	antisense	GACCAGGACGCAGAGAAGCAG
2	MP3169	sense	AAGGGGAGAAAGGGTGTCACGCTT
	MP3293	antisense	TGATGAGGCTGCCTCCACACACGTA
3	MP3169	sense	AAGGGGAGAAAGGGTGTCACGCTT
	MP3409	antisense	CTGGTGGGTGGCACAGGCAAA

Primers used Figure 4D

Supplementary Data S2

Position	Sequence
-6347	TGCCTCA
**-5394**	**TGTGTCA**
-5347	TGAATGA
**-5329**	**TGATTCA**
**-5056**	**TTAGTCA**
-4775	TGCCTCA
-4650	TGGATCA
-4641	CGCCTCAG
-4605	TGAGCCA
**-4562**	**TGACTTA**
-4543	TGGGTCA
**-4508**	**AGACTCA**
**-4301**	**TGAGTAA**
-4130	AGACTCA
**-3859**	**TGAGTCA**
-3677	TGAGGCA
-3287	TGACTCCA
-3269	TGGGTCA
**-3111**	**TGACTCA**
-3052	GGAGTCA
-2834	TGGATCA
-2370	TGAGGCA
**-1968**	**TGAAGTCA**
**-1886**	**TGAATCA**
**-1695**	**TGACTCA**
-1630	TGGGCTCA
-1388	GGACTCA
76	TGCCGCA
200	GGAGTCA
503	TGACTGA
731	TGGGTCA
2241	TGACTGC
2972	TGACACG
3608	TGACTTA
**4186**	**TGACACT**
**4240**	**TGACCCA**
4523	TAACTCA
4829	TGCGTCA
4894	TGAGGCCA
**5017**	**TGAGACA**
**5068**	**TCACGTCA**
5263	AGACTCA
**5497**	**AGAGTCA**
**5660**	**TGACTCA**
5999	TGAGTAA

Compilation of putative AP-1 DNA binding sites at the Plau locus. AP-1 sites were compiled from -6.5 kb to the end of the 3'UTR using the Jaspar (http://jaspar.cgb.ki.se/), Matinspector (http://www.genomatix.de/online_help/help_matinspector/matinspector_help.html), and Alibaba2 (http://www.gene-regulation.com/pub/programs/alibaba2/index.html) softwares. Only sites found in at least two of the databases are reported. Those found in all of them are in bold. Positions are numbered with respect to the Plau-001 TSS.

Supplementary data 3

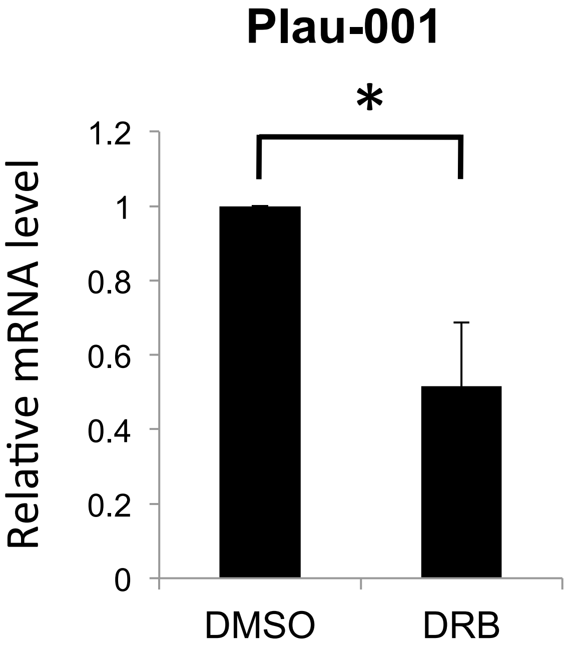

Effect of DRB on Plau-001 expression. MDA-MB231 cells were incubated for 8 hrs in presence of DRB (65μM) or DMSO as a control. RT-qPCR were carried out as in Figure 1D. Plau-001 mRNA abundance was arbitrarily set to 1 in cells incubated with DMSO for calculation of mean values. Values are the means of 3 independent experiments and error bars indicate standard deviations. The result of the Student's paired t-test is indicated on the graphs.

Supplementary data 4

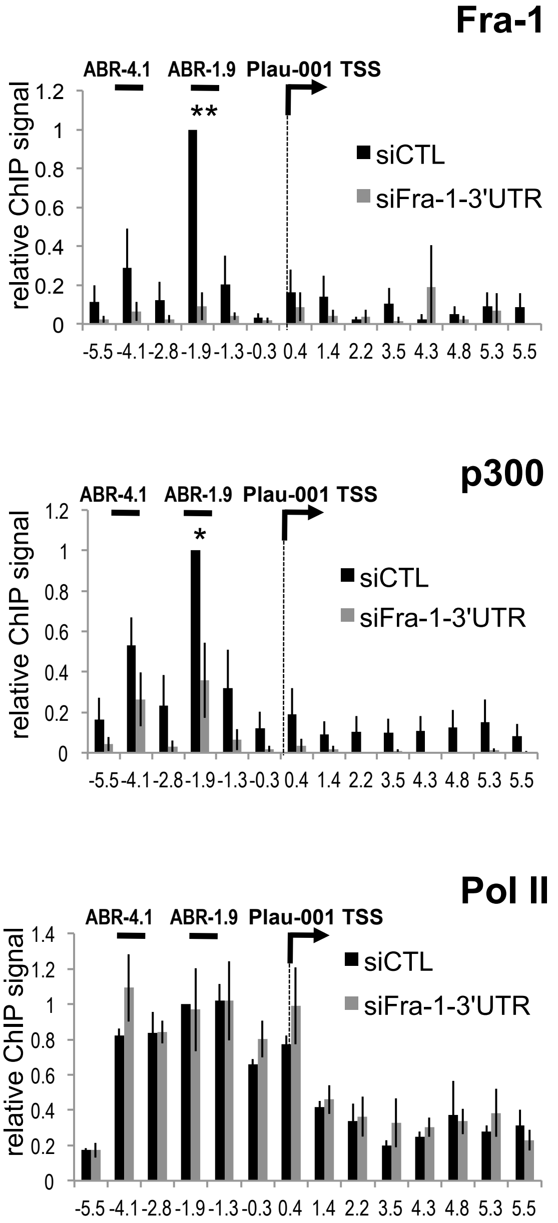

Effect of Fra-1 knockdown on the uPA locus. *Distribution of Fra-1, p300* and *Pol II on the Plau locus*. ChIPs were conducted in MDA-MB231 cells 48h after transfection of either a control siRNA (siCTL) or of the anti-Fra-1-3'UTR siRNA using specific antibodies (see Materials and Methods) directed to Fra-1, p300 or to the RPB1 subunit of Pol II. Values are the means of 3 independent experiments. Bars indicate standard deviations. Results of the Student's paired t-test are indicated on the graphs

Supplementary Data 5

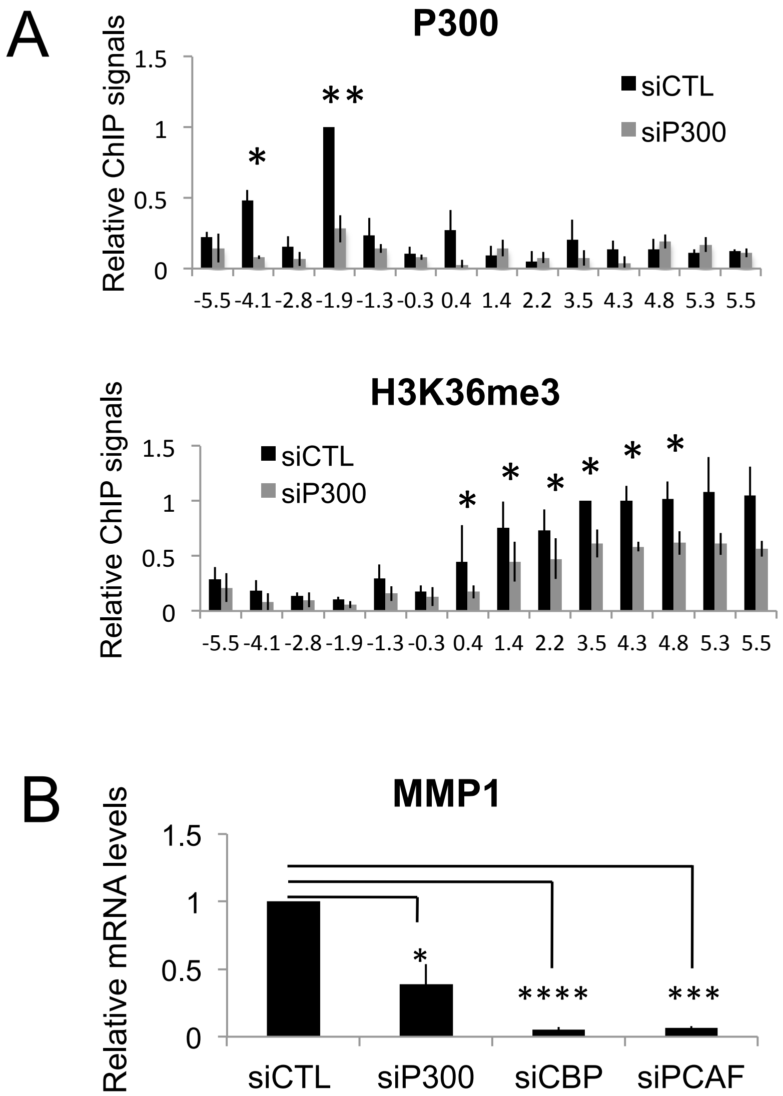

A) Effect of p300 siRNA-mediated knock down on H3K36me$_3$ levels on the uPA locus in MDA-MB231 cells. Cells were transfected for 48 hours with a siRNA directed to p300 (see Materials and Methods). ChIP experiments were conducted as described in Materials and Methods using antibodies specific for p300 (upper panel) or H3K36me3 (lower panel). Values are the means of 3 independent experiments. For mean calculations, values were normalized to that of amplicon -1.9 (upper panel) or to that of amplicon 3.5 (lower panel), arbitrarily set to 1, in cells transfected with the siCTL. Results of the Student's paired t-test are indicated on the graphs.

B) Effect of p300, CBP and pCAF on MMP1 mRNA levels. MDA-MB231 cells were transfected with either a control siRNA (siCTL) or siRNAs directed to p300, CBP ou pCAF. 48 h after transfection, RT-qPCR were carried out using the following forward and reverse primers : MP3048 TTTCCTCCACTGCTGCTGCTGCTG and MP3049 CTTTTCAACTTGCCTCCCATCATTCTTCA. MMP1 mRNA abundance was arbitrarily set to 1 in siCTL-transfected cells for calculation of mean values. Values are the mean of 3 independent experiments. Results of the Student's paired t-test are indicated on the graphs.

Supplemental Data 6

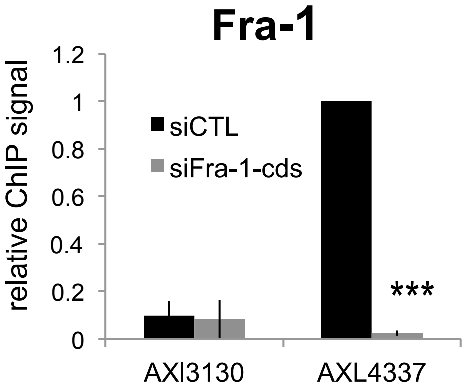

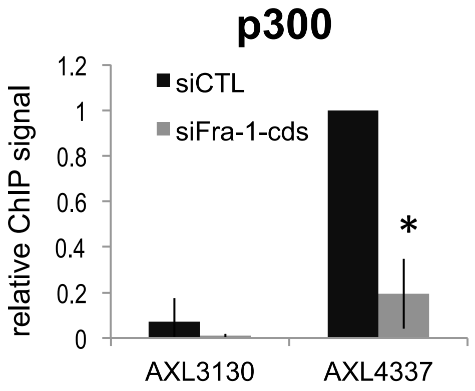

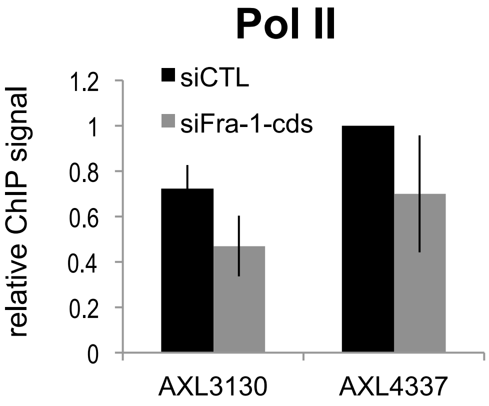

Effect of Fra-1 knockdown on the AXL locus. *Distribution of Fra-1, p300* and *Pol II on specific domains containing (AXL4337) or not (AXL3130) AP-1 sites on the AXL gene*. ChIPs were conducted in MDA-MB231 cells 48h after transfection of either a control siRNA (siCTL) or of the anti-Fra-1-cds siRNA using specific antibodies directed to Fra-1, p300 or the RPB1 subunit of Pol II. Values are the means of 3 independent experiments. Bars indicate standard deviations. Results of the Student's paired t-test are indicated on the graphs. Primers to amplify the AXL 3130 and AXL 4337 amplicons are respectively : MP3960 CTCTCCCTCCATTCACCCCGG / MP 3961 : AGGGCTGACAAAGGGGCACCA and MP3950 : GCATCAGCTTCCCGCACTCCC / MP3951: CATGCCCGATCCCCAACCCTG

Supplementary data 7

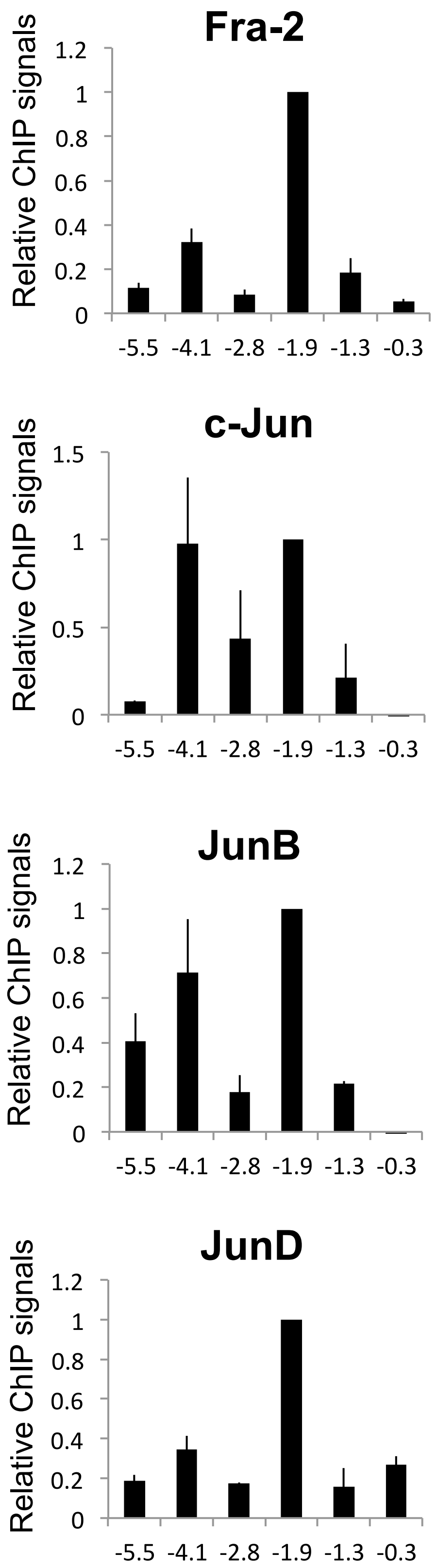

Distribution of Fra-1, c-Jun, JunB and JunD on the Plau locus. ChIP experiments were conducted in MDA-MB231 cells using antibodies specific for Fra-2 (sc-1317X, Santa Cruz Biotechnology), c-Jun (sc1694X, Santa Cruz Biotechnology), JunB (C37F9, Cell Signaling Technology) and JunD (sc74X, Santa Cruz Biotechnology) as described in Materials and Methods. Values are the mean of 2 independent experiments. For the calculations of means, all values were normalized to that of the amplicon -1.9, arbitrarily set to 1.
